# Comment on “Outcomes of Successful Versus Failed Stenting in Patients With Unilateral Atherosclerotic Renal Artery Occlusion”

**DOI:** 10.1111/jch.70186

**Published:** 2025-11-21

**Authors:** Shyam Sundar Sah, Abhishek Kumbhalwar

**Affiliations:** ^1^ Dr. D. Y. Patil Medical College Hospital and Research Centre Dr. D. Y. Patil Vidyapeeth (Deemed‐to‐be‐University) Pimpri, Pune Maharashtra India; ^2^ Dr. D. Y. Patil Dental College Hospital and Research Centre Dr. D. Y. Patil Vidyapeeth (Deemed‐to‐be‐University) Pimpri, Pune Maharashtra India

1

To the Editor:

We read with great interest the study by Li et al., which examined the long‐term outcomes of percutaneous transluminal renal angioplasty with stenting in unilateral atherosclerotic renal artery occlusion (RAO) [1]. The investigators should be commended for directly comparing successful versus failed revascularization in a well‐characterized cohort and for including dialysis‐free and event‐free survival curves over a median follow‐up period of nearly 30 months. The findings suggesting improved renal preservation after successful stenting provide valuable insights into interventional decision‐making in this challenging population. However, several methodological and interpretive issues merit consideration to contextualize these conclusions in the present study.

First, the grouping of the study by procedural outcome rather than by randomized allocation introduced a substantial baseline imbalance. Patients in the failed stenting arm had a lower estimated glomerular filtration rate (eGFR) and a higher renal resistive index (RI) before the intervention, both markers of irreversible parenchymal injury [2]. This selection bias may have exaggerated the apparent protective effect of successful stenting. A propensity‐weighted or covariate‐adjusted survival analysis could clarify whether the observed differences in major adverse cardiovascular or renal events (MACRE) truly reflect procedural benefits rather than baseline disease severity. Clinically, this distinction determines whether stenting should be viewed as restorative or merely prognostic in nature.

Second, the study defined renal benefit through serum creatinine and eGFR changes without accounting for single‐kidney function. Since unilateral RAO often coexists with compensatory hyperfiltration of the contralateral kidney, global eGFR improvements may not represent the recovery of the treated kidney [3]. Incorporating split‐renal scintigraphy or duplex‐derived flow indices at follow‐up would better delineate parenchymal rescue versus contralateral adaptation, which has implications for patient selection and counseling in the future.

Third, reliance on the composite MACRE endpoint from the Cardiovascular Outcomes in Renal Atherosclerotic Lesions (CORAL) trial may have limited clinical specificity. Death, myocardial infarction, and renal replacement therapy (RRT) capture distinct pathophysiological trajectories. Parsing cardiovascular events from renal components could reveal whether stenting mainly delays dialysis initiation or modifies systemic vascular risk [4]. Translationally, this distinction affects whether the intervention should be considered renal protective or cardiorenal integrative therapy.

Finally, the potential predictive value of preprocedural RI, which is markedly elevated in failed cases, warrants formal validation. Integrating RI thresholds with dynamic imaging modalities, such as blood‐oxygen‐level‐dependent magnetic resonance, could enable a reproducible pre‐stenting viability algorithm. Such a framework would advance beyond retrospective observation toward precision revascularization in ischemic nephropathy.

In summary, while the study provides encouraging data on the potential benefits of successful stenting in unilateral atherosclerotic RAO, the lack of adjustment for baseline functional disparity and the absence of renal‐specific outcome metrics limit causal inference. Future multicenter, prospective analyses incorporating single‐kidney physiology and standardized viability criteria are needed to define which patients derive durable renal and cardiovascular benefits from the intervention.

## Funding

The authors have nothing to report.

## Ethics Statement

The authors have nothing to report.

## Conflicts of Interest

The authors declare no conflicts of interest.

## Generative AI Use Statement

Paperpal and ChatGPT 5, were utilized solely for language, grammar, and stylistic refinement. These tools had no role in the conceptualization, data analysis, interpretation of results, or substantive content development of this manuscript. All intellectual contributions, data analysis, and scientific interpretations remain the sole work of the authors. The final content was critically reviewed and edited to ensure accuracy and originality. The authors take full responsibility for the accuracy, originality, and integrity of the work presented.

## Data Availability

The authors have nothing to report.
